# Long-term efficacy of infliximab for refractory ulcerative colitis: results from a single center experience

**DOI:** 10.1186/1471-230X-14-80

**Published:** 2014-04-23

**Authors:** Satoshi Yamada, Takuya Yoshino, Minoru Matsuura, Naoki Minami, Takahiko Toyonaga, Yusuke Honzawa, Yoshihisa Tsuji, Hiroshi Nakase

**Affiliations:** 1 Department of Gastroenterology and Hepatology, Graduate School of Medicine, Kyoto University, 54 Kawahara-cho, Shogoin, Sakyo-ku, Kyoto 606-8507, Japan; 2 The Third Department of Internal Medicine, Kansai Medical University, Osaka, Japan

**Keywords:** Ulcerative colitis, Infliximab, Immunomodulator, Infliximab intensification

## Abstract

**Background:**

The long-term efficacy of infliximab (IFX) for patients with refractory ulcerative colitis (UC) is unclear. The aim of this study was to assess the long-term outcomes of IFX treatment in patients with refractory UC.

**Methods:**

Thirty-three patients with refractory UC who received IFX treatment at Kyoto University Hospital between 2003 and 2013 were retrospectively evaluated. IFX intensification was defined as a dose escalation (up to 10 mg/kg) and/or shorter intervals between infusions (every 4–6 weeks).

**Results:**

Of the 33 patients who received scheduled infusions of IFX, 24 (72.7%) achieved clinical remission within 8 weeks after initiating IFX treatment. Of these 24 responders, 17 (70.8%) experienced a relapse of UC and required IFX intensification, and 16 (66.7%) eventually maintained clinical remission with IFX treatment, including IFX intensification. Of the 33 patients, 6 (18.2%) underwent colectomy during IFX treatment. Multivariate regression analysis showed that a serum C-reactive protein (CRP) concentration <5 mg/L two weeks after starting IFX was a predictor of a positive clinical response to IFX induction therapy. No severe adverse events occurred in UC patients treated with IFX.

**Conclusion:**

IFX intensification was necessary for long-term maintenance of remission and to prevent colectomy in patients with refractory UC.

## Background

Ulcerative colitis (UC) is a relapsing inflammatory bowel disease of the colon that often requires long-term therapy to maintain remission [1]. Although most patients are successfully managed with mesalamine formulations, approximately 25% of patients fail these or other therapies and require treatment with immunomodulators, including infliximab (IFX), cyclosporin, and/or tacrolimus, and/or colectomy [2]. IFX (Remicade: Janssen, Malvern, PA) is a chimeric monoclonal immunoglobulin G1 (IgG1) antibody against tumor necrosis factor (TNF)-α that binds with high affinity to free and membrane-bound TNF-α and neutralizes its biologic activity [3]. In patients with UC, IFX downregulates TNF-α in the colonic mucosa and is associated with reduced histologic inflammation [4].

Large randomized controlled trials examining the effects of IFX in patients with UC, as well as several cohort studies, have reported that short- to medium-term IFX is safe and effective for inducing and maintaining remission in patients with UC and Crohn’s disease (CD) [1,5-10]. One year clinical remission rates were found to be 35% in patients with UC [5] and 33% in patients with CD [10]. Some patients with CD, however, do not respond to IFX, whereas others experience a loss of efficacy over time or become intolerant to the drug [11]. Some CD patients who lose the ability to respond to IFX require more intensive treatment to maintain remission, such as an IFX dose escalation or shorter intervals between infusions [11]. Similarly, 60% of UC require IFX escalation [11-13].

The effects of immunomodulators, such as azathioprine (AZA) and mercaptopurine (MP), on clinical outcomes in UC patients treated with IFX are unclear. Steroid-free remission rates were reported higher in AZA-naïve UC patients treated with IFX and AZA than in patients treated with either agent [14]. Concomitant administration of an immunomodulator and an anti-TNF-α agent, however, is not appropriate for long-term therapy, because a high proportion of CD patients treated with an immunomodulator experience adverse events [15]. Since the effects of combined treatment with IFX and an immunomodulator on long-term clinical outcomes in UC patients are unclear, we retrospectively analyzed long-term clinical outcomes of UC patients following IFX induction treatment.

## Methods

### Patients

This study enrolled 33 patients with refractory UC who underwent IFX treatment at Kyoto University Hospital from January 2003 to June 2013. UC was diagnosed based on the result of endoscopy and pathologic examination. IFX induction therapy, consisting of 5 mg/kg IFX at 0, 2, and 6 weeks, was followed by scheduled maintenance IFX treatment every 8 weeks thereafter [5]. All subjects provided informed consent. The study protocol conformed to the Declaration of Helsinki and was approved by the Institutional Review Boards at Kyoto University Hospital.

### Definitions

Refractory UC was defined as steroid-resistant, steroid-dependent, or refractory to immunosuppressive therapies. Steroid-resistant refractory UC was defined the lack of a clinical response to a systemic daily dose of ≧30 mg of prednisolone over at least 2 weeks. Steroid-dependent refractory UC was defined as a failure to taper prednisolone below 10 mg/day within 12 weeks or relapse within 12 weeks after prednisolone discontinuation. Immunosuppressive refractory UC was defined as the lack of a clinical response to tacrolimus, even at trough levels of 10 to 15 ng/ml; or the lack of a clinical response to the thiopurine agents AZA and MP, at the doses adjusted to achieve white blood cell counts between 3000 and 5000/μL or 6-thioguanine nucleotide (6-TGN) concentrations between 235 and 450 pmol/8 × 10^8^ erythrocytes. Disease activity of UC was evaluated according to a modified Truelove and Witts severity Index (MTWSI) score. Patients were categorized as either responders or nonresponders to IFX based on global assessments by gastroenterologists within 8 weeks of IFX initiation. Clinical remission was defined as an MTWSI score lower than 4 within 8 weeks after initiating IFX therapy. Intensification of IFX treatment was defined as a dose escalation (up to 10 mg/kg) and/or a shorter interval between infusions (every 4–6 weeks). Treatment with thiopurine agents was optimized based on serum 6-TGN concentrations, with some patients requiring the addition of allopurinol to increase their serum 6-TGN levels. At each examination, patients were assayed for cytomegalovirus (CMV) infection by immunohistochemistry, CMV antigenemia, and quantitative real-time polymerase chain reaction using colonic biopsy specimens (mucosal PCR) to detect CMV-DNA in colonic mucosa. Concomitant CMV infection was defined as positive on at least one of these assays, as described by the guidelines of the European Crohn’s and Colitis Organization [16]. Serum hemoglobin and albumin concentrations were measured at IFX initiation, and serum C-reactive protein (CRP) concentrations were measured immediately (0 week) and 2 weeks after the first administration of IFX. Mucosal healing was defined as a Mayo endoscopic score of 0 or 1 [5].

### Assessments

The long-term efficacy of IFX treatment in patients with refractory UC was determined by evaluating the remission-maintenance rate and the colectomy-free rate of the 24 responders to IFX treatment including IFX intensification. Since mucosal healing, which is associated with reduced rates of hospitalization and colectomy, was found to contribute to long-term clinical outcomes in patients with UC, we also evaluated the mucosal healing rate in responders to IFX treatment. To evaluate the importance of mucosal healing in the clinical course of IFX-treated UC patients, we compared colectomy rates in patients who did and did not achieve mucosal healing. Finally, we evaluated the efficacy of combinations of IFX and an immunomodulator in patients with refractory UC by comparing remission-maintenance and the colectomy-free rates in patients receiving combination therapy and those receiving IFX monotherapy. Remission-maintenance and mucosal healing rates in patients who responded to IFX induction treatment were evaluated at 6, 12, 24 and 36 months after IFX initiation, whereas colectomy-free rates was evaluated at 3, 6, 12 and 36 months. Moreover, factors predictive of clinical remission to IFX induction therapy were evaluated by assessing the correlations of remission with MTWSI score, CMV negative status, serum albumin >35 g/L at IFX initiation, serum CRP concentration at IFX initiation, and CRP concentration <5 mg/L two weeks after IFX initiation.

### Statistical analysis

Continuous variables were analyzed using Student’s *t*-test if normally distributed or Wilcoxon’s rank sum test if the data were nonparametric. Categorical variables were analyzed using Pearson’s chi-squared test or Fisher’s exact test if any cell number was less than 5. A *p* level of 0.05 was considered statistically significant. The cumulative colectomy-free and remission-maintenance rates were assessed using the Kaplan-Meier method, and groups were compared using the log-rank test stratified by study. Predictive factors were analyzed by multivariate statistics. Statview software was used for all statistical analysis.

## Results

### Patient characteristics

The 33 patients with UC consisted of 20 men and 13 women, of mean age 43.2 years (range 17-75 years) and mean disease duration at start of IFX treatment of 7.0 years (range, 0.5-29 years; Table 1). Their mean MTWSI score was 9.4 points (range, 6–18 points), with all 33 patients having moderate to severe symptoms, and their mean Mayo endoscopic score was 2.8 points (range, 2–3 points). Twenty patients (60.6%) had extensive colitis, with the remaining 13 (39.4%) having left-sided colitis. Twenty-nine patients (87.9%) were steroid-dependent or steroid-refractory, while the other 4 patients (12.1%) were refractory to immunomodulators such as methotrexate and tacrolimus. Upon the initiation of IFX treatment, 29 patients (87.9%) were treated with a 5-aminosalicylic acid formulation, 11 (33.3%) were treated with corticosteroids, 16 (48.5%) were treated with concomitant thiopurine, and 13 (39.4%) were treated with concomitant tacrolimus. Biopsy specimens from inflammatory mucosa of 11 patients (33.3%) were positive for CMV-DNA, with two of these eleven patients treated with anti-viral agents before starting IFX treatment. Twenty-five patients (75.8%) were non-smokers and eight (24.2%) were smokers.

**Table 1:** Demographic and clinical characteristics of UC patients

**Characteristics**	**All patients n = 33**
Sex (men/women), n (%)	20 (60.6)/13 (39.4)
Age (years)*	43.2 ± 17.4
Disease duration (years)*	7.0 ± 5.7
Modified Truelove and Witts severity index*	9.4 ± 3.2
Mayo score (endoscopy)*	2.8 ± 0.4
Extent of disease	
Left-side type, n (%)	13 (39.4)
Extensive colitis, n (%)	20 (60.6)
Concomitant medications	
5-ASA formulation, n (%)	29 (87.9)
Corcicosteroids, n (%)	11 (33.3)
Azathioprine/Mercaptopurine, n (%)	16 (48.5)
Tacrolimus, n (%)	13 (39.4)
Cytomegalovirus, n (%)	11 (33.3)

*IFX*: infliximab, *5-ASA*: 5-aminosalicylic acid.

Results reported as number (%) of patients or as *mean ± standard deviation.

### Clinical course of UC patents after IFX induction treatment

**Figure 1:**
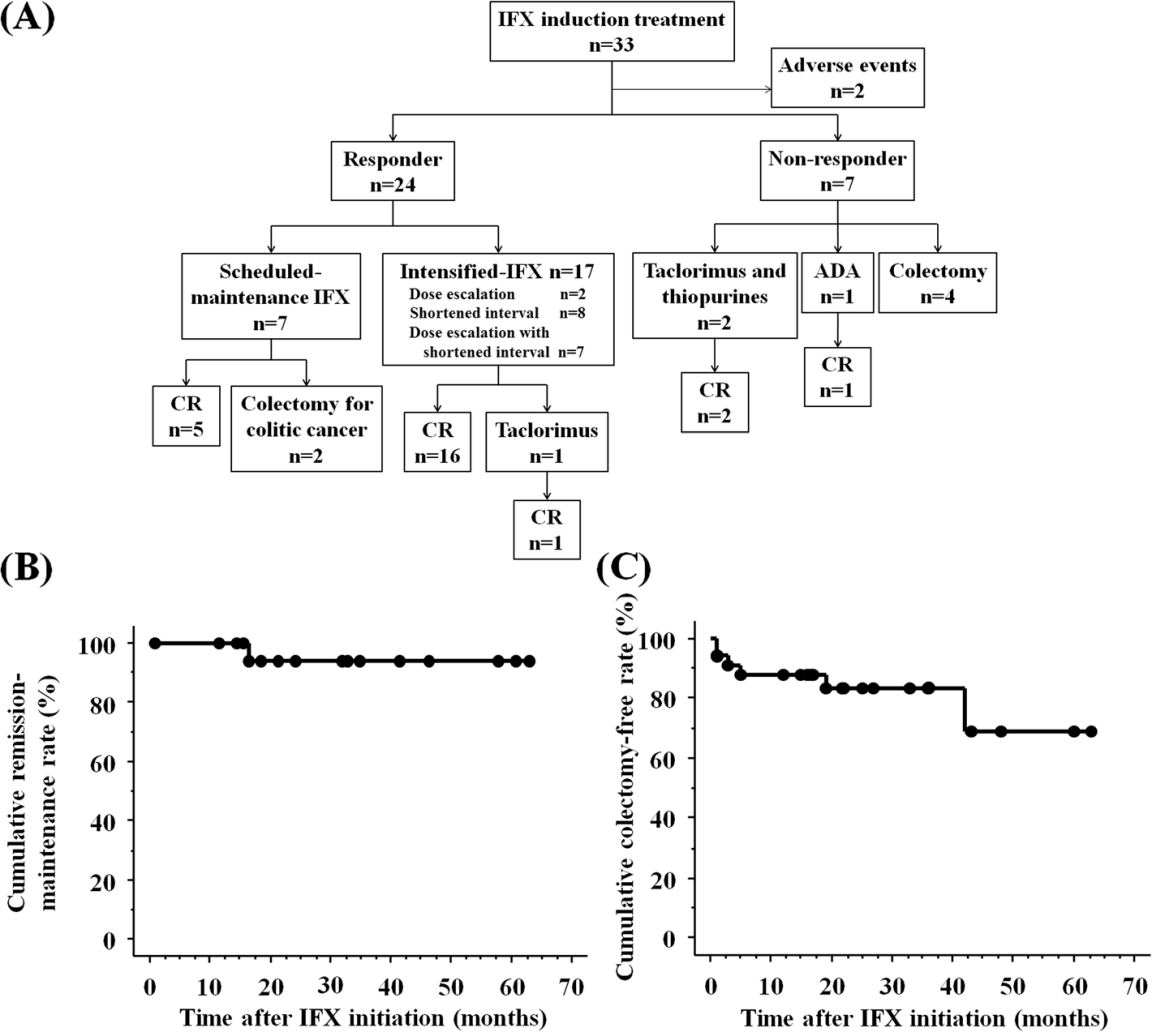
**Clinical course and survival curves of UC patients treated with IFX. (A)** Clinical course of UC patients who received IFX induction treatment. **(B)** Cumulative remission maintenance rate in 24 responders to IFX maintenance treatment, including IFX intensification. **(C)** Cumulative colectomy-free rate in patients who received IFX treatment during follow up.

Of the 33 patients, 31 (93.9%) were able to continue IFX induction treatment, whereas the other two (6.1%) experienced adverse events requiring discontinuation of IFX induction therapy (Figure 1A). Following the initiation of IFX induction therapy, 24 of 31 patients (77.4%) responded and proceeded to IFX scheduled maintenance treatment, whereas seven (22.6%) did not respond to IFX. Of the 24 responders, seven (29.2%) maintained clinical remission on IFX maintenance therapy, whereas 17 (70.8%) experienced a relapse of UC and required IFX intensification. IFX intensification consisted of dose escalation in two, shortened intervals between doses in eight, and a combination of the two in seven. The median duration of IFX maintenance treatment in 17 responders was 3.0 months (range, 1-40 months) and their median time to relapse after IFX induction was 3.0 months (range, 1-34 months). After IFX intensification, 16 patients (94.1%) achieved and maintained clinical remission, whereas one patient (5.9%) required tacrolimus owing to failure of IFX intensification. The remission maintenance rates 6, 12, 24 and 36 months after IFX initiation in the 24 responders who received IFX maintenance treatment were 100.0% (22/22), 100.0% (21/21), 92.3% (12/13) and 90.0% (9/10), respectively. Based on Kaplan-Meier analysis, the cumulative remission-maintenance rate of the 24 responders to IFX maintenance treatment including IFX intensification was estimated to be 90.9% at 63 months (Figure 1B), indicating the importance of IFX intensification for UC patients who have flares during IFX maintenance treatment.

Of the 33 patients who received IFX induction treatment, six (18.2%) ultimately underwent colectomy during follow-up (Figure 1A), including four who did not respond to IFX induction treatment and two who were found to have colon cancer during scheduled IFX maintenance treatment. Based on Kaplan-Meier analysis, the colectomy-free rate at 3, 6, 12, and 36 months after IFX initiation were 92.9% (26/28), 88.5% (23/26), 82.6% (19/23) and 64.3% (9/14), respectively (Figure 1C).

### Mucosal healing in patients that received IFX maintenance treatment including IFX intensification

Of the 24 responders, 17 (70.8%) underwent colonic examinations at a median 10.0 months (range, 1–39 months) after IFX initiation to assess mucosal healing after achievement of clinical remission. Of these 17 patients, 13 (76.5%) achieved mucosal healing, including 11 receiving IFX maintenance treatment and six receiving IFX intensification therapy. In contrast, the remaining 4 patients (23.5%) did not show mucosal healing, despite the absence of clinical symptoms. Rates of mucosal healing 6, 12, 24 and 36 months after IFX initiation were 87.5% (7/8), 80.0% (8/10), 78.6% (11/14) and 81.3% (13/16), respectively. None of the patients who showed mucosal healing group underwent colectomy, whereas two of the four patients (50.0%) who did not show mucosal healing underwent colectomy for colon cancer, a difference that was statistically significant (*p* = 0.007).

### Efficacy and safety of combinations of IFX and an immunomodulator

**Figure 2:**
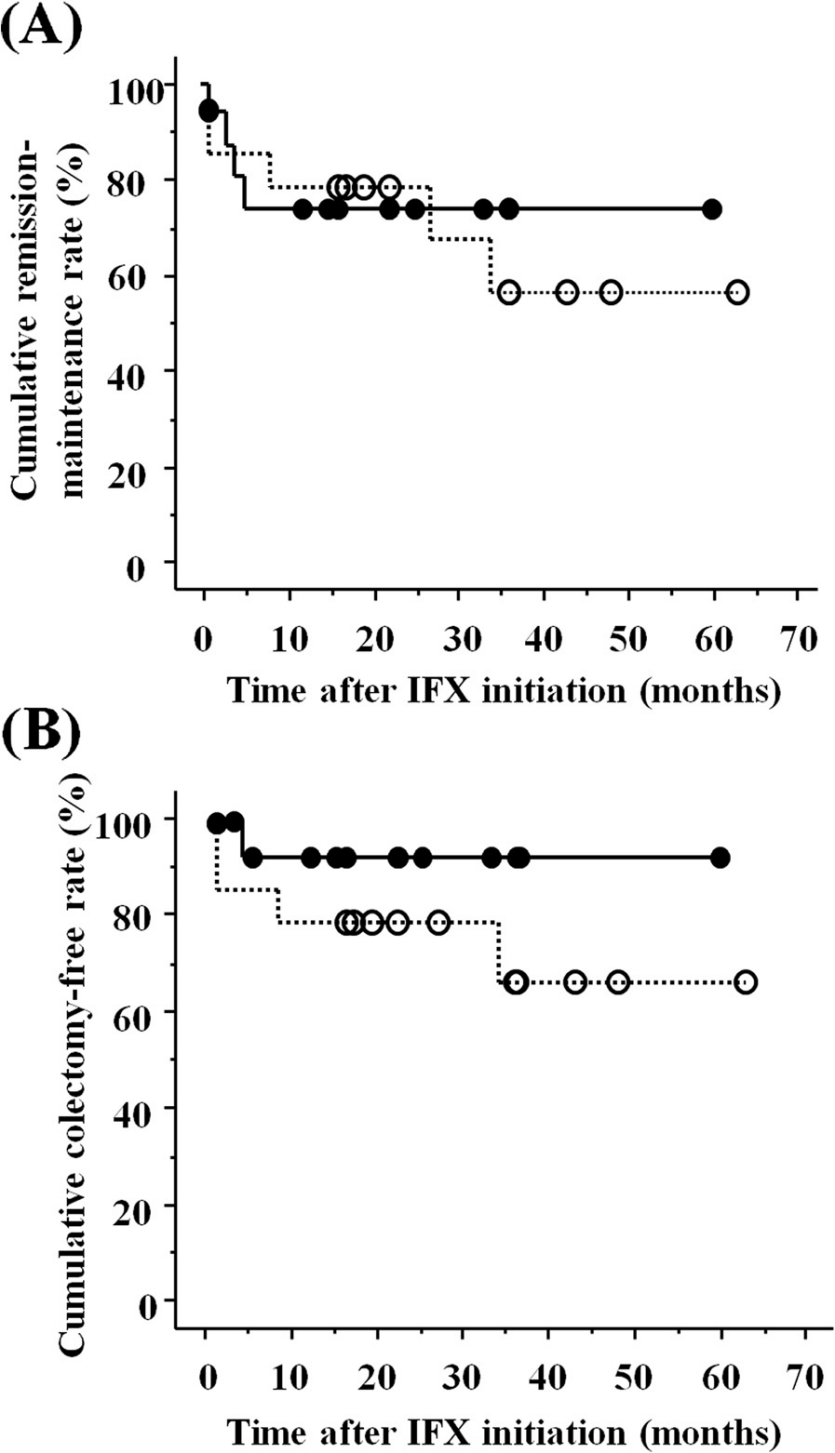
**Survival curves between in patients receiving IFX and immunomodulators and IFX alone. (A)** Cumulative remission-maintenance rates in patients receiving combination therapy (solid line) and IFX monotherapy (dotted line). Rates were similar in these two groups (73.9% at 60 months vs 56.1% at 63 months, *p* = 0.74). **(B)** Cumulative colectomy-free rates in patients receiving combination therapy (solid line) and IFX monotherapy (dotted line). These rates were also similar (91.7% at 60 months vs 66.2% at 63 months, *p* = 0.21).

Of the 33 patients who received IFX induction treatment, 16 (48.5%) received combination therapy and 17 (51.5%) received IFX monotherapy during follow-up. Clinical remission rates in these two groups were 75.0% (12/16) and 70.6% (12/17), respectively. The remission maintenance rates at 6, 12, 24 and 36 months after IFX initiation were 66.7% (10/15), 66.7% (10/15), 58.3% (7/12) and 37.5% (3/8), respectively, in patients who received combination therapy, and 81.3% (13/16), 75.0% (12/16), 69.2% (9/13) and 60.0% (6/10), respectively, in patients who received IFX monotherapy. The cumulative remission maintenance rates were similar in the combination and monotherapy groups (73.9% at 60 months *vs* 56.1% at 63 months, *p* = 0.74; Figure 2A). Colectomy rates in these two groups were 11.7% (2/16) and 23.5% (4/17), respectively. The colectomy-free rates 6, 12, 24 and 36 months after IFX initiation were 91.7% (11/12), 91.7% (11/12), 90.0% (9/10) and 87.5% (7/8), respectively, in patients who received combination therapy, and 82.4% (14/17), 76.5% (13/17), 66.7% (8/12) and 60.0% (6/10), respectively, in patients who received IFX monotherapy. The cumulative colectomy-free rates were similar in the combination and monotherapy groups (91.7% at 60 months *vs* 66.2% at 63 months, *p* = 0.21; Figure 2B).

No adverse events were observed in UC patients who received combinations of IFX and an immunomodulator, whereas two patients treated with IFX alone experienced infusion reactions related to IFX.

### Factors associated with the clinical response to IFX induction treatment

The demographics and clinical characteristics of patients in the responder and nonresponder groups were similar (Table 2). The percentage of responders with serum CRP concentration <5 mg/L 2 weeks was significantly higher than that of nonresponders (*p* = 0.006). Multivariate logistic regression analysis (Table 3) showed that serum CRP <5 mg/L 2 weeks after initiating IFX treatment was a positive predictor of a clinical response to IFX treatment (odds ratio 8.86, *p* = 0.08).

**Table 2:** Demographic and clinical characteristics of responders and non-responders to IFX induction treatment

**Characteristics**	**Responders (n = 24)**	**Non-responders (n = 7)**	***p *** **value**
Sex (men/women), n (%)	16 (66.7)/8 (33.3)	3 (42.9)/4 (57.1)	0.26
Age (years)	41.8 ± 17.1	48.0 ± 17.8	0.43
Disease duration (years)*	6.5 ± 4.1	9.6 ± 9.2	0.23
Modified Truelove and Witts severity index*	9.7 ± 3.2	10.6 ± 3.4	0.57
Mayo score (endoscopy)*	2.8 ± 0.4	2.9 ± 0.3	0.58
Extent of disease			
Left-side type, n (%)	12 (50.0)	1 (14.3)	0.09
Extensive colitis, n (%)	12 (50.0)	6 (85.7)	
Concomitant medications			
Corticoteroids, n (%)	9 (37.5)	1 (14.3)	0.25
Azathioprine/Mercaptopurine, n (%)	11 (45.8)	4 (57.1)	0.60
Tacrolimus, n (%)	8 (33.3)	4 (57.1)	0.26
Cytemegalovirus, n (%)	6 (25.0)	4 (57.1)	0.17
Serum hemoglobin level >9.8 g/dl at IFX initiation, n (%)	16 (66.7)	2 (28.6)	0.07
Serum albumin level >35 g/L at IFX initiation, n (%)	12 (50.0)	1 (14.3)	0.21
Serum CRP level <5 mg/L at 2 weeks after IFX initiation, n (%)	18 (75.0)	1 (14.3)	0.006

*IFX*: infliximab, *CRP*: C-reactive protein.

Results reported as number (%) of patients or *mean ± standard deviation.

**Table 3:** Multivariate analysis of factors predicting clinical remission in response to IFX induction therapy (5 mg/kg)

	***p *** **value**	**Odds ratio**	**95% CI**
Modified Truelove and Witts severity index	0.27	0.81	0.54–1.20
Negative for cytomegalovirus	0.26	1.08	0.77–19.17
Serum albumin level >35 g/L at IFX initiation	0.27	6.37	0.21–190.12
Serum CRP level at IFX initiation	0.64	1.08	0.78–1.49
Serum CRP level <5 mg/L at 2 weeks after IFX initiation	0.08	8.86	0.59–132.23

*IFX*: infliximab, *IM*: immunomodulator, *CRP*: C-reactive protein, *CI*: confidence interval.

### Factors associated with IFX intensification

We also evaluated the clinical and demographic characteristics associated with the need for IFX intensification. The characteristics of patients who maintained clinical remission with IFX maintenance treatment and those with IFX intensification were similar, except for their Mayo endoscopic score and concomitant use of corticosteroids (Table 4).

**Table 4:** Demographic and clinical characteristics of the patients who maintained clinical remission or experienced a relapse of UC with IFX scheduled-maintenance treatment

**Characteristics**	**Clinical remission with IFX maintenance (n = 7)**	**Relapse requiring IFX intensification (n = 17)**	***p *** **value**
Sex (men/women), n (%)	5 (71.4)/2 (28.6)	11 (64.7)/6 (35.3)	0.64
Age (years)*	38.6 ± 20.7	43.1 ± 15.2	0.64
Disease duration (years)*	7.2 ± 3.5	6.8 ± 4.3	0.68
Modified Truelove and Witts severity index*	7.9 ± 3.6	10.3 ± 3.0	0.23
Mayo score (endoscopy)*	3.0 ± 0.0	2.7 ± 0.5	0.02
Extent of disease			
Left-side type, n (%)	2 (28.6)	10 (58.8)	0.17
Extensive colitis, n (%)	5 (71.4)	7 (41.2)	
Concomitant medications			
Corticoteroids, n (%)	0 (0.0)	7 (41.2)	0.04
Azathioprine/Mercaptopurine, n (%)	4 (57.1)	9 (52.9)	0.85
Tacrolimus, n (%)	3 (42.9)	5 (29.4)	0.53
Hemoglobin >9.8 g/dl at IFX initiation, n (%)	5 (71.4)	10 (58.8)	0.56
Serum albumin >35 g/L at IFX initiation, n (%)	2 (28.6)	10 (58.8)	0.18
Serum CRP <5 mg/L at 2 weeks after IFX initiation, n (%)	6 (85.7)	12 (70.6)	0.44

*IFX*: infliximab, *CR*: clinical remission, *CRP*: C-reactive protein.

Result reported as number (%) of patients or *mean ± standard deviation.

### Clinical outcome of UC patients with CMV reactivation

Eleven of the 33 patients (33.3%) experienced CMV reactivation before IFX treatment, with eleven being positive for CMV-DNA in colonic tissue, three positive by immunohistochemistry and five positive by antigenemia. Two of these 11 patients (18.2%) were treated with anti-viral agents before initiating IFX treatment. Of these 11 patients, six patients (54.5%) achieved clinical remission after initial IFX induction therapy, whereas five (45.5%) did not. The induction remission rate was lower in CMV-positive than in CMV-negative patients (54.5 vs. 81.8%), although the difference was not statistically significant. Among CMV-positive patients, the five non-responders to IFX had a significantly higher disease activity index (MTWSI) and a significantly higher rate of concomitant tacrolimus use at the initiation of IFX than the six responders to IFX (Table 5), indicating that CMV reactivation could affect the therapeutic efficacy of IFX in patients with refractory UC.

**Table 5:** Demographic and clinical characteristics of the responders and non-responders of IFX with CMV infection

**Characteristics**	**Responders with CMV infection (n = 6)**	**Non-responders with CMV infection (n = 5)**	***p *** **value**
Sex (men/women), n (%)	4 (66.7)/2 (33.3)	2 (40.0)/3 (60.0)	0.38
Age (years)*	50.8 ± 16.2	41.4 ± 17.2	0.42
Disease duration (years)*	4.5 ± 4.8	10.2 ± 9.6	0.32
Modified Truelove and Witts severity index*	8.3 ± 1.7	12.4 ± 2.2	0.02
Mayo score (endoscopy)*	2.8 ± 0.4	3.0 ± 0.0	0.37
Extent of disease			
Left-side type, n (%)	2 (33.3)	0 (0.0)	0.15
Extensive colitis, n (%)	4 (66.7)	5 (100.0)	
Concomitant medications			
Corticoteroids, n (%)	0 (0.0)	1 (20.0)	0.25
Azathioprine/Mercaptopurine, n (%)	3 (50.0)	2 (40.0)	0.74
Tacrolimus, n (%)	1 (16.7)	4 (80.0)	0.04

*IFX*: infliximab, *CR*: clinical remission, *CRP*: C-reactive protein.

Result reported as number (%) of patients or *mean ± standard deviation.

### Adverse events

Of the 33 patients, two (6.1%) experienced adverse events, including anaphylactic shock and drug eruption in one patient each (Figure 1A). Anaphylactic shock occurred at the first IFX infusion and drug eruption occurred at the second IFX infusion. Both patients were switched from IFX to adalimumab, with clinical remission achieved and maintained with this agent.

## Discussion

Despite the large number of UC patients treated with IFX, limited data are available on the long-term effects of IFX treatment for UC in clinical practice. The present study was therefore designed to evaluate the long-term efficacy of IFX in refractory UC patients with a median follow-up of almost 3 years.

We found that more than half the responders to initial IFX therapy required IFX intensification owing to symptom relapse. These findings are similar to those of studies showing high rates of IFX escalation therapy (shorter infusion cycles and/or higher doses) in UC patients during maintenance treatment [5,7,17]. The present of patients showing a short-term response to IFX has been reported to range from 33 to 73% [5,7-9,18]. The reason for the relatively higher short-term response rate in our patient cohort remains unclear, although the concomitant use of tacrolimus in more than 30% patients receiving IFX induction therapy may have affected our results.

Our findings also showed that IFX intensification can maintain clinical improvement in patients with refractory UC, over a median follow-up of 1.5 years. We found that 70.8% (17/24) of the initial responders to IFX required IFX intensification and that 87.5% (21/24) maintained clinical remission. Additionally, all UC patients who received IFX intensification therapy avoided colectomy, with a cumulative colectomy-free rate in our 33 IFX-treated patients being 64.8% at 63 months. Previous reports have shown that the percentage of patients with refractory UC requiring IFX escalation ranges from 14 to 54% [7,9,17-20], although we observed a higher percentage. Although secondary loss of response to IFX may be owed to the generation of antibodies to IFX or differences in clearance, it may also be by differences among studies in IFX intensification (escalation) ratios. Cesarini, et al. reported that clinical remission was maintained by 68.3% of UC patients 52 weeks after IFX intensification; of these patients, 36.6% had received IFX-dose escalation and 63.4% received IFX infusions at shorter intervals, with none receiving both [20]. In contrast, the clinical remission rate in patients who required IFX intensification was 93.3% at week 52; of the latter, 47.0% had received IFX-dose escalation, 11.8% received IFX infusions at shorter intervals, and 41.2% received both higher doses of IFX and doses at shortened intervals. These data suggested that differences between studies in remission rates of UC patients receiving IFX intensification may be because of differences in the percentage of UC patients who received both higher dose of IFX and doses at shortened intervals.

The combination of IFX and an immunomodulator has been reported superior to IFX alone in inducing remission in UC patients [14]. The long-term differences in clinical outcomes, however, have not yet been confirmed. Although the cumulative rates of remission-maintenance (73.9% at 60 months *vs* 56.1% at 63 months, *p* = 0.74) and colectomy-free status (91.7% at 60 months *vs* 66.2% at 63 months, *p* = 0.21) were higher in patients receiving combinations of IFX and an immunomodulator than in patients receiving IFX monotherapy, these differences were not statistically significant. The discrepancy between our and previous results may be owed to differences in patient characteristics, because all patients enrolled in our study had refractory UC.

Identifying factors predictive of the efficacy of IFX in patients with refractory UC is clinically important. Among the factors previously reported to predict clinical responses to IFX treatment in UC patients are hemoglobin concentration, serum albumin concentration, disease activity, normalization of serum CRP concentration after IFX induction, and the trough of IFX [7,9,17,21-23]. We found that serum CRP concentration <5 mg/L 2 weeks after initiating IFX was predictive of a clinical response to IFX in patient with refractory UC. Biomarkers such as CRP, fecal calprotectin, and fecal lactoferrin have been reported to act as surrogate markers of mucosal inflammation in patients with inflammatory bowel disease [23-25], with CRP being a particularly sensitive marker in UC patients. Thus, early reduction of serum CRP may be a useful marker for evaluating the efficacy of IFX treatment.

CMV reactivation in inflamed mucosa may contribute to the exacerbation of UC [26,27]. However, the therapeutic effects of anti-viral treatment of UC patients with CMV reactivation have not been evaluated because virological criteria identifying patients who require anti-viral treatment have not been established. We found that the remission rate was lower in UC patients with CMV reactivation than in CMV-negative patients, suggesting a reduction of CMV reactivation in the colonic mucosa may affect the efficacy of immunomodulatory treatment. Although responses to IFX therapy were reported unaffected by HCMV infection/disease [28], an algorithm for the management of CMV reactivation includes antiviral treatment in IBD patients with high CMV load in tissue (>250 copies/mg) [29]. Thus, further studies are required to determine the effects of CMV infection on UC patients treated with IFX.

Mucosal healing has been suggested as a treatment goal in patient with UC [5,7,30], with the percentages of patients achieving mucosal healing in response to IFX ranging from 45 to 53%, and 43% in refractory UC patients [7]. We observed mucosal healing in 76.5% (13/17) of UC patients receiving IFX maintenance treatment, including IFX intensification. Thus, the ability of IFX intensification to enhance mucosal healing rates suggests a favorable clinical outcome.

The present study has several limitations. First, mucosal healing was not evaluated by colonoscopy in all enrolled patients, and mucosal healing assessment time varied widely. In addition, multivariate analysis could not be performed to identify factors predicting the need for IFX intensification because of the small number of patients enrolled in the study. Furthermore, some treatments drugs available in Japan to moderate or severe UC are not available in western countries including cytapheresis, tacrolimus, adalimumab and IFX. Finally, this study was a single center analysis, suggesting the need for larger, multicenter studies to evaluate the effects of long-term IFX treatment in patients with refractory UC.

## Conclusions

In conclusion, we found that 64.8% of our patients with refractory UC avoided colectomy by receiving IFX treatment. IFX was more effective when given in combination with thiopurines than when administered as monotherapy. IFX-intensification treatment, however, was required by 70.8% of UC patients. IFX dose should be tailored to individual patients, based on factors baseline serum TNF-α concentration, in the treatment of refractory UC.

## Abbreviations

IFX: Infliximab; UC: Ulcerative colitis; TNF: Tumor necrosis factor; CD: Crohn’s disease; AZA: Azathioprine; MP: Mercaptopurine; 6-TGN: 6-thioguanie nucleotide; MTWSI: Modified Truelove and Witts Severity Index; CMV: Cytomegalovirus; DNA: Deoxyribonucleic acid; CRP: C-reactive protein.

## Competing interests

The authors declare that they have no competing interests.

## Authors’ contributions

SY, TY, MM and HN designed the study. SY performed the major role of collecting patients’ data and wrote the manuscript. NM, TT, YH TY, MM and HN collected patients’ data and were involved in editing the manuscript. YT performed the statistical analysis. All authors read and approved the final manuscript.

## Pre-publication history

The pre-publication history for this paper can be accessed here:

http://www.biomedcentral.com/1471-230X/14/80/prepub
